# *In Vivo* Antimalarial Activity of* Annona muricata* Leaf Extract in Mice Infected with* Plasmodium berghei*

**DOI:** 10.1155/2016/3264070

**Published:** 2016-03-22

**Authors:** Voravuth Somsak, Natsuda Polwiang, Sukanya Chachiyo

**Affiliations:** Department of Clinical Chemistry, Faculty of Medical Technology, Western University, Kanchanaburi 71170, Thailand

## Abstract

Malaria is one of the most important infectious diseases in the world. The choice for the treatment is highly limited due to drug resistance. Hence, finding the new compounds to treat malaria is urgently needed. The present study was attempted to evaluate the antimalarial activity of the* Annona muricata* aqueous leaf extract in* Plasmodium berghei* infected mice. Aqueous leaf extract of* A. muricata* was prepared and tested for acute toxicity in mice. For efficacy test* in vivo*, standard 4-day suppressive test was carried out. ICR mice were inoculated with 10^7^ parasitized erythrocytes of* P. berghei* ANKA by intraperitoneal injection. The extracts (100, 500, and 1000 mg/kg) were then given orally by gavage once a day for 4 consecutive days. Parasitemia, percentage of inhibition, and packed cell volume were subsequently calculated. Chloroquine (10 mg/kg) was given to infected mice as positive control while untreated control was given only distilled water. It was found that* A. muricata* aqueous leaf extract at doses of 100, 500, and 1000 mg/kg resulted in dose dependent parasitemia inhibition of 38.03%, 75.25%, and 85.61%, respectively. Survival time was prolonged in infected mice treated with the extract. Moreover, no mortality to mice was observed with this extract up to a dose of 4000 mg/kg. In conclusion, the* A. muricata* aqueous leaf extract exerted significant antimalarial activity with no toxicity and prolonged survival time. Therefore, this extract might contain potential lead molecule for the development of a new drug for malaria treatment.

## 1. Introduction

Malaria, a tropical parasitic disease, is one of the most important infectious diseases in the world. Estimated 3.3 billion of the total world population live in areas with malaria risk and an estimated death of 660,000. Africa is the most affected continent, accounting for about 90% of all malaria deaths [1]. This disease is caused by* Plasmodium* species and transmitted by the bite of female* Anopheles* mosquito. The global strategy for malaria mainly focuses on case management through provision of drugs capable of reducing or eliminating parasites. However, multiple antimalarial drug resistant* Plasmodium* parasites and the emergence of insecticide resistant* Anopheles* mosquitoes are causing not only the spread of malaria to new areas but also its reemergence in areas where it had previously been eradicated [2]. In addition, chloroquine resistance now occurs throughout the whole world [3]. Therefore, the search for new antimalarial, either synthetic or natural, is important for killing of malaria parasite. The use of medicinal plant extracts for the treatment of malaria has a long and successful tradition [4]. For example, quinine was isolated from* Cinchona* and artemisinin from Qinghaosu [5]. This explains why a lot of current researches focus on natural and plant-derived products as they can be sourced easily, can be locally available, and can be selected on the basis of their pharmacological use [6, 7].

*Annona muricata*, a member of the Annonaceae family, is now widely distributed throughout tropical and subtropical parts of the world such as South and North America, Africa, Asia, and Southeast Asia including Thailand [8].* A. muricata* extract presents antioxidant, anti-inflammation, antimicrobials, antiparasitic, antidiabetes, antihyperlipidemia, hepatoprotective, and anticancer activities [9–14]. Extensive phytochemical evaluations of* A. muricata* extract have shown the presence of various compounds, including alkaloids, polyphenols, flavonoids, essential oils, cyclopeptides, and kaempferol [15]. Moreover,* A. muricata* has been shown to be a generally rich source of acetogenin compounds that are active compounds in this plant extract [8, 15]. Furthermore, the leaf extract of* A. muricata* was assayed against* P. falciparum* and showed promising antimalarial effect [12, 16]. However, antimalarial activity of this plant extract against* P. berghei* infected mouse model has not yet been reported. Hence, the present study aimed at evaluating the* in vivo* antimalarial activity of* A. muricata* aqueous leaf extract against* P. berghei* infected mice.

## 2. Materials and Methods

### 2.1. Preparation of Aqueous Leaf Extract

The dried leaves of* Annona muricata* were purchased from Royal Project Foundation shop, Chiang Mai, Thailand. The dried leaves were ground using electric blender, and extraction was then carried out by dispersing 10 g of the dried powdered plant material in 100 mL of distilled water (DW). Microwave method was used at 360 W for 5 min and incubated at room temperature for 24 h [17]. Filtration was then performed through Whatman number 1 filter paper and followed by lyophilization. Aqueous leaf extract of* A. muricata* was stored at −20°C until used. Before experiments, the extract was then dissolved in DW at appropriate doses.

### 2.2. Acute Toxicity Test

Acute toxicity of* A. muricata* aqueous leaf extract was carried out using modified Lorke's method [18]. Fifteen female ICR mice were randomized into 5 groups of three mice each and were then given orally with 100, 500, 1000, 2000, and 4000 mg/kg. The mice were observed for signs of toxicity such as paw licking, salivation, stretching of the entire body, weakness, sleep, respiratory distress, coma, and death in the first four hours and subsequently daily for 7 days. The oral median lethal dose was calculated using the following formula:

(1) LD50=Minimum  toxic  dose×Maximum  tolerate  dose.

### 2.3. Experimental Mice

Pathogen-free (bacteria, fungi, and parasites), 4-week-old female ICR (Imprinting Control Region) mice weighting 25–30 g were obtained from the National Laboratory Animal Center, Mahidol University, Bangkok, Thailand, and kept for at least one week with sterile-filtered tap water and pellet diet (CP diet 082, Perfect Companion Company, Bangkok, Thailand)* ad libitum*. Experiments were started in 5-6-week-old mice. Procedures of the animal experiments were ratified by the Ethical Committee on Animal Experimentation, Faculty of Medical Technology, Western University.

### 2.4. Rodent Malaria Parasite

*Plasmodium berghei* ANKA (PbANKA) was used. Frozen parasites from stock were passaged at least once through ICR mice before experiments. Parasitemia was daily monitored by microscopy of Giemsa stained thin blood smear. The percentage of parasitemia (% parasitemia) was calculated using the formula

(2) % parasitemia=Number  of  parasitized  erythrocytesNumber  of  erythrocytes×100.

When the parasitemia showed 15–20%, infected mouse blood was then collected by cardiac puncture and suspended in phosphate buffer saline (PBS). Infection for experiments was carried out by intraperitoneal (IP) injection of approximately 10^7^ parasitized erythrocytes in mice.

### 2.5. Determination of Mean Survival Time

Mortality was monitored daily and the number of days from the time of inoculation of the parasite up to death was recorded for each mouse. The mean survival time (MST) was calculated as follows:

(3) MST=Sum  of  survival  time  of  all  mice  in  a  groupTotal  number  of  mice  in  that  group days.

### 2.6. Determination of Packed Cell Volume

The packed cell volume (PCV) of each mouse was measured by collecting tail blood into heparinized hematocrit tubes, sealed with Critoseal, and placed in a hematocrit centrifuge at 10,000 rpm for 5 min. PCV was then read using hematocrit reader according to the following formula:

(4) $$\begin{array}{c} PCV=\frac{Volume\;\;of\;\;packed\;\;erythrocytes}{Total\;\;volume\;\;of\;\;blood}\times 100. \end{array}$$

### 2.7. Standard Antimalarial Drug

Chloroquine (CQ) was used as standard antimalarial drug in this study. The drug at chosen dose was freshly prepared in DW and administered orally by gavage. Drug dose, expressed in mg/kg of body weight, was adjusted at the time of administration according to the weight of the mice.

### 2.8. Suppressive Test

The standard 4-day suppressive test against PbANKA infection in mice was employed [19]. Naïve ICR mice were inoculated by IP injection of 10^7^ parasitized erythrocytes. The infected mice were randomly divided into 5 groups of 5 mice per group and treated for 4 consecutive days with 100, 500, and 1000 mg/kg of extract orally by gavage. Two control groups were used: the positive control was treated daily with 10 mg/kg of CQ while the untreated group was given DW. On day 5 of experiment, parasitemia and percentage of inhibition were calculated according to the following formula:

(5) $$\begin{array}{c} \%\;inhibition=\frac{Parasitemia\;\;of\;\;untreated\;\;group-Parasitemia\;\;of\;\;extract\;\;treated\;\;group}{Parasitemia\;\;of\;\;untreated\;\;group}\times 100. \end{array}$$

### 2.9. Statistics

The one-way ANOVA was used to analyze and compare the results at a 95% confidence. Values of *p* < 0.05 were considered significant. Results were expressed as mean ± standard error of mean (SEM).

## 3. Results

### 3.1. Acute Toxicity

The results of the acute toxicity evaluation of* A. muricata* aqueous leaf extract showed no remarkable behavioral changes in the extract administered mice. No mortality occurred within the observation period of 7 days. However, behavioral signs of toxicity were observed in mice given 4000 mg/kg which include paw licking, salivation, and stretching and reduce activity. There was however no mortality at all the doses used. The oral median LD_50_ was estimated to be ≥2500 mg/kg.

### 3.2. Effect of* A. muricata* Aqueous Leaf Extract on Parasitemia and Mean Survival Time

The results of the standard 4-day suppressive test of the* A. muricata* aqueous leaf extract on parasitemia and survival time in PbANKA infected mice were summarized in Table 1. The* A. muricata* aqueous leaf extract presented dose dependent antimalarial effect against PbANKA infection in mice and caused a significant (*p* < 0.05) inhibition when compared to the untreated group. The highest inhibition (85.61%) was observed with the dose of 1000 mg/kg. However, the mice were not completely cured from the infection in all treatment doses but did significantly (*p* < 0.05) prolong the mean survival time at all dose levels.

### 3.3. Effect of* A. muricata* Aqueous Leaf Extract on Packed Cell Volume

The effect of* A. muricata* aqueous leaf extract on PCV on day 0 and day 4 comparison was indicated in Table 2. The extract showed protective effect on PCV reduction in dose dependent manner, although the dose of 100 mg/kg resulted in significant (*p* < 0.05) reduction of PCV.

## 4. Discussion

Medicinal plants contain active constituents that have potential for medicinal use and pharmaceutical drug companies make use of these plants [7, 20, 21]. The extract of* A. muricata* was reported to contain different classes of metabolites such as tannins, alkaloids, flavonoids, polyphenols, saponins, diterpenoids, essential oils, kaempferol, and acetogenin compounds [8, 15, 22, 23]. Diterpenoids, flavonoids, polyphenols, saponins, alkaloids, kaempferol, and acetogenin were known to have antimalarial activity [24–27]. Moreover, antioxidant effect in this plant may also contribute to the antimalarial activity. It has been reported that antioxidant activity can inhibit heme polymerization as heme has to be oxidized before polymerization, and the unpolymerized heme is very toxic for malaria parasite [28, 29]. In addition, these active compounds in this plant extract may be acting singly or in synergy with one another to exert the observation antimalarial activity. All doses of the extract displayed significant and dose dependent inhibition in comparison with untreated control. So, the activity may be due to the individual or synergistic effect of the metabolites found in the extract like alkaloid, flavonoids, polyphenols, saponins, kaempferol, and acetogenin. It is finding similar with the previous study that showed antimalarial activity against* P. falciparum* [12, 16].

Scoring system used to assess signs of toxicity in mice has not been performed. Physical observation for signs of toxicity in mice has just been done. From this study, all mice treated with this extract were normal in physical activities and survived. Blood biochemical tests in order to ensure the toxicity have not been carried out. However, in this observation signs of toxicity have been used in other studies [18].Interestingly,* A. muricata* aqueous leaf extract did not show mortality within 7 days up to a dose of 4000 mg/kg indicating that this extract is safe. In general, if the lethal dose of the test compound is 3 times more than the minimum effective dose, the compound is considered a good candidate for further studies [30, 31]. The result is in agreement with other studies done on the same plant with crude extracts, indicating that no death was observed with different dose levels, 1000, 3000, and 4000 mg/kg [12, 16]. However, abnormalities like depression, weakness, and rough hair coat were observed with 4000 mg/kg aqueous crude leaf extract of* A. muricata*.

The* A. muricata* aqueous leaf extract prolonged the MST of the infected mice indicating that it suppressed PbANKA and reduced the overall pathologic effect of the parasite on the infected mice. Moreover, infected mice suffer from anemia because of erythrocyte destruction, either by malaria multiplication or by spleen reticuloendothelial cell action [32]. In this study, the extract showed protection against PCV reduction as compared to day 0 indicating that antioxidant and antimalarial effects of this extract may play a critical role. So, the* A. muricata* aqueous leaf extract inhibited and reduced parasitemia in mouse followed by protection of PCV reduction resulting to prolong the MST of the infected mice.

## 5. Conclusions

It is evident based on these findings that the oral administration of* A. muricata* aqueous leaf extract (100–1000 mg/kg) to mice for 4 days significantly reduced parasitemia of PbANKA in experimental mice with nontoxicity. The implication of this finding is that* A. muricata* aqueous leaf extract possesses potent antimalarial effect and may therefore serve as potential sources of safe, effective, and affordable antimalarial drugs. The displayed high* in vivo* antimalarial property and lack of toxic effect render* A. muricata* a candidate for the bioassay-guided isolation of compounds which could develop into new lead structures and candidates for drug development programs against human malaria.

## Figures and Tables

**Table 1:** Effect of *A. muricata* aqueous leaf extract on parasitemia and mean survival time.

Treatment	Dose (mg/kg)	% parasitemia	% inhibition	Mean survival time
*A. muricata*	100	34.02 ± 1.5	38.03^*∗*^	18.32 ± 0.84^*∗*^
	500	13.59 ± 1.8	75.25^*∗∗*^	24.79 ± 1.76^*∗∗*^
	1000	7.9 ± 0.2	85.61^*∗∗∗*^	28.75 ± 1.37^*∗∗∗*^

Chloroquine	10	0.52 ± 0.05	99.05	30.33 ± 1.61

Distilled water	1 mL	54.9 ± 4.39	0	7.79 ± 0.73

^*∗*^ *p* < 0.05, ^*∗∗*^ *p* < 0.01, and ^*∗∗∗*^ *p* < 0.001, compared to untreated control. Parasitemia and mean survival time were expressed as mean ± SEM, *n* = 5. Chloroquine: positive control; distilled water: untreated control.

**Table 2:** Effect of *A. muricata* aqueous leaf extract on packed cell volume.

Treatment	Dose (mg/kg)	Packed cell volume	
		Day 0	Day 4
*A. muricata*	100	53.24 ± 2.73	40.14 ± 3.26^*∗*^
	500	50.56 ± 3.12	48.22 ± 3.18
	1000	53.71 ± 2.59	52.15 ± 1.76

Chloroquine	10	51.55 ± 3.14	52.47 ± 2.27

Distilled water	1 mL	52.15 ± 3.25	38.26 ± 2.31^*∗∗*^

^*∗*^ *p* < 0.05 and ^*∗∗*^ *p* < 0.01, compared to day 0. Packed cell volume was expressed as mean ± SEM, *n* = 5. Chloroquine: positive control; distilled water: untreated control.
